# Metastatic cutaneous apocrine adenocarcinoma successfully treated with systemic anti‐androgen therapy—A case report

**DOI:** 10.1002/ccr3.3434

**Published:** 2020-10-30

**Authors:** Fanny Collette, Marc Hamoir, Pascal Van Eeckhout, Philippe D’Abadie, Thierry Duprez, Sandra Schmitz, Jean‐Pascal Machiels

**Affiliations:** ^1^ Institut Roi Albert II Department of Medical Oncology Cliniques universitaires Saint‐Luc and Institut de Recherche Clinique et Expérimentale (POLE MIRO) Université catholique de Louvain Brussels Belgium; ^2^ Institut Roi Albert II Department of Head and Neck Surgery Cliniques universitaires Saint‐Luc and Institut de Recherche Clinique et Expérimentale (POLE MIRO) Université catholique de Louvain Brussels Belgium; ^3^ Institut Roi Albert II Department of Pathology Cliniques universitaires Saint‐Luc Brussels Belgium; ^4^ Institut Roi Albert II Department of Nuclear Medicine Cliniques universitaires Saint‐Luc and Institut de Recherche Clinique et Expérimentale (POLE MIRO) Université catholique de Louvain Brussels Belgium; ^5^ Institut Roi Albert II Department of Medical Imaging Cliniques universitaires Saint‐Luc Brussels Belgium

**Keywords:** androgen receptor, anti‐androgen therapy, metastatic cancer, Primary cutaneous apocrine adenocarcinoma, sweat glands

## Abstract

Primary cutaneous apocrine adenocarcinoma (PCAC) is an extremely rare neoplasm involving the sweat glands. Due to a lack of cases, there is no consensus for the systemic treatment of locally advanced or metastatic PCAC. Anti‐androgen therapy may have activity in inoperable or metastatic PCAC with high androgen receptor (AR) expression.

## Graphical abstract

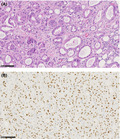

## 1 INTRODUCTION

Primary cutaneous apocrine adenocarcinoma (PCAC) is an extremely rare type of adnexal tumor of the skin. It usually occurs in areas of high sweat gland density such as the axilla, the groin, and anogenital regions, but can occur anywhere else on the skin.^1^, ^2^, ^3^ Only 25 cases have been described up to now involving the modified apocrine glands of the eyelid, also called Moll's glands.^4^, ^5^

The diagnosis of PCAC is challenging and involves both the pathologist and the clinician. The tumor usually presents as painless, indurate, reddish purple papules, nodules, or plaques. The typical histological features of apocrine differentiation are abundant granular eosinophilic cytoplasm with luminal decapitation secretion.^1^ Such features may easily be confused with a cutaneous metastasis originating from a breast apocrine carcinoma. Some immunohistochemistry can be helpful in distinguishing these two distinct entities, such as adipophilin, estrogen receptor (ER), progesterone receptor (PgR), human epidermal receptor 2 (HER‐2), cytokeratin 5/6, p63, GATA3, GCDFP‐15, and mammaglobin.^6^, ^7^, ^8^

The clinical behavior of PCAC is variable, from relatively indolent to highly aggressive,^3^ depending on the histological grade (well, moderate, or poorly differentiated). Most cases are localized at the time of diagnosis, but metastatic dissemination occurs in approximately 30% of cases^9^ and is often associated with regional lymph node invasion.

Due to limited number of reported cases and the availability of only retrospective data, there is no consensus regarding treatment. The usually accepted treatment is wide surgical excision with clear margin, and lymphadenectomy in the event of regional lymph node invasion. The role of lymphadenectomy is less clear if the regional lymph nodes are not involved. Adjuvant treatments, such as radiotherapy or chemotherapy, can be used in patients with poor prognostic factors (poorly differentiated tumor, lymph node invasion, or positive surgical margins), although their effectiveness has not been validated in randomized trials.^2^, ^3^, ^10^, ^11^, ^12^, ^13^, ^14^

For patients with distant metastases, there is a clear lack of data with regards to the systemic treatment of PCAC. A few case reports have demonstrated clinical benefit for some chemotherapeutic agents (anthracyclines, taxanes, platinum compounds), targeted therapies (trastuzumab, pertuzumab, lapatinib—if there is HER‐2 overexpression and/or amplification), and endocrine therapies (tamoxifen or letrozole—if the tumor expresses estrogen receptors).^12^, ^24^

We report here the case of a patient with metastatic PCAC successfully treated with enzalutamide. To the best of our knowledge, this is the first case report of metastatic PCAC treated with anti‐androgen therapy.

## 2 CASE REPORT

A 68‐year‐old Caucasian male was first diagnosed in November 1994 with an apocrine adenocarcinoma of the left upper eyelid involving the Moll's gland (cT2cN0M0 according to the TNM 8th Edition). The tumor was treated surgically with a wide left upper palpebral excision and immediate palpebral plasty by rotation of a temporal skin flap. The surgical margins were clear.

The patient was followed closely and developed several local and/or regional relapses in 1999, 2000, 2003, and 2016 which were treated with surgery and radiotherapy (Table 1). Moreover, he developed a progressive functional loss of the left eye due to chronic postradiation keratopathy, leading to an enucleation on March 2017. There was no histological sign of malignancy.

**Table 1:** Medical history of the patient case reported

Date	Localization	Surgical treatment	Adjuvant treatment
January 1999	Local recurrence at the left internal palpebral angle, with invasion of the orbit	Wide surgical excision of the left orbital internal angle and reconstruction with a glabellar skin flap (R0)	Brachytherapy 60 Gy within 6 d
September 2000	Regional recurrence in the left (zones II and III) and right (zone II) jugulo‐carotid area, the left sub‐mandibular area (zone I), and the left parotid area	Left parotidectomy with bilateral cervical lymphadenectomy (zones I to III) (R0, 4 positive nodes out of 34)	External beam radiotherapy 64 Gy within 45 d
June 2003	Local recurrence at the left external palpebral angle	Wide surgical excision (R2)	Brachytherapy 60 Gy within 4 d
November 2016	Cutaneous metastases along the left parotidectomy scar and on the left lateral cervical area	Wide superficial excision of the left parotidectomy scar and the cervical budding lesion, with reconstruction using an anterolateral thigh (ALT) free flap (R1)	/
March 2017	No neoplastic recurrence	Left eye enucleation (chronic postradiation keratopathy and functional loss of the left eye)	/

Abbreviation: Gy, Gray.

The patient remained disease‐free for one year following the last surgical resection. However, in June 2018, some new, reddish purple spots appeared around the cervical skin flap. ^18^FDG‐PET‐CT (positron emission tomography) and MRI (magnetic resonance imaging) confirmed the presence of a neoplastic recurrence in the left orbit above the ocular prosthesis, and a suspect skin thickening in front of the horizontal branch of the left mandible. There were also multiple bone metastases. His blood test was normal which included a PSA level of 1.26 μ g/L (normal value < 2.5 μg/L).

**Figure 1:**
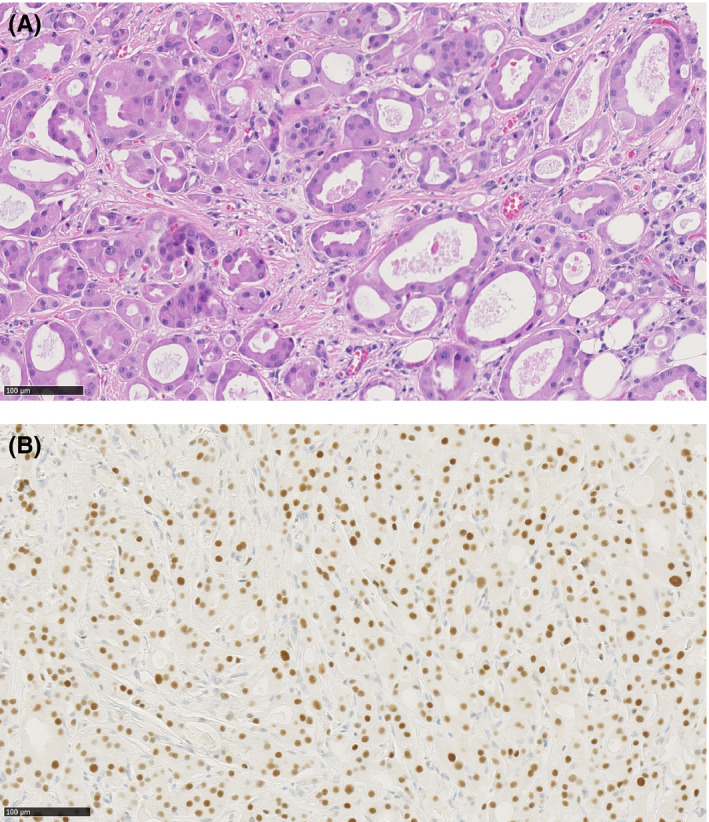
Biopsy of the cervical lesion in HE (hematoxylin‐eosin) and in IHC (anti‐androgen receptor antibody in immunohistochemistry). A, HE: Infiltrative neoplasm composed of apocrine tubular structures with lumen formation. Decapitation secretion is seen. B, IHC: Immunohistochemistry shows a strong and diffuse nuclear positivity for androgen receptor

The biopsy of the cutaneous lesion showed tumor proliferation characterized by glandular structures in cribiform clusters with abundant eosinophilic cytoplasm, which confirmed the cutaneous metastasis of the known apocrine adenocarcinoma (Figure 1A). The immunohistochemical profile showed strong androgen receptor (AR) expression (100% staining of tumor cells; Figure 1B) and GCDFG‐15 expression, but there was no expression of ER, PgR, or mammaglobin. HER‐2 was positive in immunohistochemistry (2 to 3+), but was not amplified in CISH (chromogenic in situ hybridization). Target gene sequencing was performed (26 genes studied). With regards to an actionable genomic alteration, a mutation was observed in the PTEN gene (p.Asp393*), which was corroborated by the absence of PTEN expression in the immunohistochemistry.

As the tumor was relatively indolent with slow growth and no visceral metastasis, and our patient had a good performance status (Eastern Cooperative Oncology Group (ECOG) = 0), we decided to treat him with the anti‐androgen enzalutamide, which had proven efficacy in metastatic prostate cancer.^25^

On August 10, 2018, enzalutamide 160 mg per day was started with the approval of our ethics committee and the patient, who signed an informed consent given the treatment's “non‐standard” nature. The patient was seen three weeks later; there were no adverse events, and the plaques around the skin flap were already less extended and less reddish.

**Figure 2:**
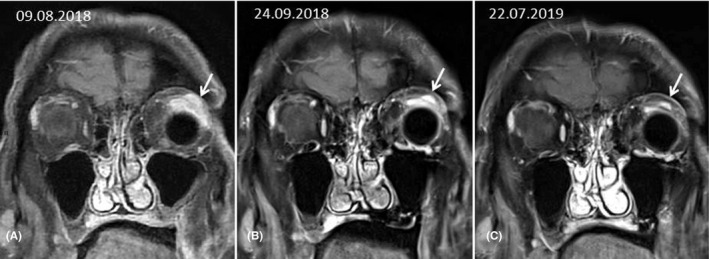
Serial MR monitoring of therapeutic response: coronal contrast‐enhanced T1‐weighted views with fat saturation option in similar slice location. A, Baseline before treatment initiation showing enhanced tumoral mass (arrow) in the lateral upper quadrant of the left orbit including the lacrymal gland and the superior oblique and superior rectus extra‐ocular muscles (EOMs). The eyeball is a prosthesis. B, Six weeks later the tumor has shrunk and the EOMs have become more delineable. C, Delayed control at one year showing complete recovery with normalized anatomy except decreased enhancement within the lacrymal gland featuring scaring fibrosis. Additional view. Oblique sagittal contrast‐enhanced T1‐weighted view with fat saturation option showing a tumoral mass of 15 mm in maximal axis involving the full thickness of the eyelid including tarsal plate and eyelid margins

**Figure 3:**
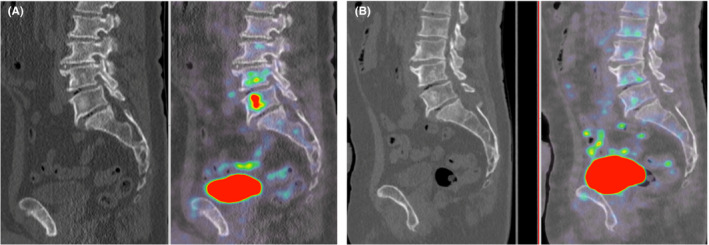
A 2′‐deoxy‐2′‐[18F] fluoro‐D‐glucose positron emission tomography at baseline. B. 2′‐deoxy‐2′‐[18F] fluoro‐D‐glucose positron emission tomography 3 mo after enzalutamide initiation

Seven weeks after treatment initiation, an ^18^FDG‐PET‐CT showed an almost complete metabolic response of the left orbital lesion and of the bone metastases. Figures 2 and 3, for example, show morphologic and metabolic response in the left orbit and the 5th lumbar vertebra (SUV max: 6.1 versus SUV max: 1.9 three months after starting enzalutamide).

At 19 months of treatment with enzalutamide (still 160 mg per day), a complete metabolic response was confirmed according to ^18^FDG‐PET‐CT and the patient maintained excellent tolerance to ongoing treatment.

## 3 DISCUSSION

Primary cutaneous apocrine adenocarcinoma (PCAC) is an extremely rare neoplasm involving the sweat glands of the skin. To date, the largest series of PCAC was reported by Hollowell et al in 2012,^2^ who retrospectively identified 186 patients over a 33‐year period (from 1973 to 2006) through the American SEER program (National Cancer Institute Surveillance, Epidemiology and End Results). The incidence of PCAC was estimated at 0.0049 to 0.0173 per 100,000 patients per year. The median age was 67 years.

Due the limited number of reported cases, prognostic factors are difficult to establish, but the histopathological grade seems to be useful. The Bloom‐Richardson grading system, as used in breast cancer,^1^, ^13^ has been suggested to help define treatment. Indeed, as shown by Robson *et al* in a series of 24 patients,^1^ there was a statistically significant relationship between overall survival and the Bloom‐Richardson grade: median survival was two years for grade 3 tumors, and not achieved for grade 1 and 2 tumors after a median follow‐up of two years (*P* = .0007). Therefore, patients with a low‐grade tumor could be treated with wide surgical excision without any adjuvant therapy.^13^ Lymph node invasion, which was present in 40% to 69% of cases at the time of diagnosis according to different series,^2^, ^13^, ^14^ is also associated with worse outcomes as are positive surgical margins and distant metastases.

In the largest retrospective series currently reported, the median overall survival was 51.5 months but 55 months for localized disease, 33 months for positive lymph node disease, and 14.5 months in the metastatic setting. Local recurrence seems to be more frequent than distant metastases. The most common sites for distant metastases are bone, lung, pleura, liver, brain, kidney, and, more rarely, other visceral organs.^2^

For localized disease, wide surgical excision with clear margins (1‐2 cm) is generally considered the cornerstone of treatment based upon validated guidelines for similar cutaneous lesions.^3^, ^10^, ^11^ In the event of regional lymph node invasion, lymphadenectomy is usually recommended. The role of lymphadenectomy is less clear in the absence of regional lymph node involvement. As regional lymph node metastasis occurs at a high rate and is associated with worse outcomes, some authors suggest systematic lymphadenectomy.^12^ Others recommend carrying out a sentinel lymph node biopsy to guide treatment strategy (like in melanoma), with lymph node dissection reserved for those with a positive biopsy result.^2^

There is no consensus regarding the role of adjuvant radiotherapy. It can be offered for large (>5 cm) or moderate to poorly differentiated tumors with lymphovascular space invasion, those with positive margins (<1 cm), or in the event of wide lymph node invasion (>4 nodes involved and/or extranodal extension).^10^, ^11^, ^12^, ^21^, ^26^ Adjuvant chemotherapy has been proposed by some investigators for patients with poor prognostic factors using adjuvant breast cancer regimens.^15^, ^20^

Currently, there is no standardized systemic treatment for locally advanced or metastatic disease, and only a few case reports are available in the literature. As it is not possible to conduct prospective clinical trials for such a rare pathology, and given the similarities with apocrine breast carcinoma, most reported cases have been treated with standard breast cancer regimens. Anthracycline‐based chemotherapy has been given in a few cases,^15^, ^16^ followed or not by oral fluorinated pyrimidine.^17^ Taxanes (docetaxel, paclitaxel) have also been used,^12^, ^15^, ^18^ associated or not with a platinum compound (cisplatin, carboplatin) and radiotherapy.^19^, ^20^, ^21^, ^22^ For patients with HER‐2 amplification, treatment has used targeted therapies (trastuzumab, pertuzumab or lapatinib)^18^, ^22^, possibly in association with chemotherapy.^15^, ^23^ Endocrine therapies, such as tamoxifen or letrozole, have also been used in patients with ER + tumors.^12^, ^21^ A patient whose tumor was positive for RANK‐L was successfully treated with denosumab in addition to chemoradiation,^19^ and a patient with PD‐L1 positive staining in about 60% of tumor cells was successfully treated with pembrolizumab.^24^

It can be difficult to distinguish primary cutaneous apocrine carcinoma, which involves the sweat glands of the skin, from a cutaneous metastasis of apocrine breast carcinoma, which refers to a subtype of breast cancer showing histological features of apocrine differentiation.

Apocrine breast carcinoma expresses the androgen receptor (AR). Three phase II studies investigated the activity and safety of agents targeting the androgen receptor in molecular apocrine breast carcinoma (MABC), meaning that the selection was based on the expression of AR in immunohistochemistry (IHC), and not on histological features of apocrine differentiation (Table 2).^27^, ^28^, ^29^

**Table 2:** Activity of anti‐androgen therapy in molecular apocrine breast cancer

Reference	Population	Treatment	N	% AR	ORR	CBR	Median PFS	OS
Gucalp et al 2013^27^	ER negative PgR negative	Bicalutamide	24	>10%	0%	At 6 mo: 19% [95% CI, 7% – 39%]	12 weeks [95% CI, 11 ‐ 22 wk]	Not reported
Bonnefoi et al 2016^28^	ER negative PgR negative HER‐2 negative	Abiraterone acetate	30	≥10%	6.7% (95% CI 0.8%–22.1%)	At 6 mo: 20% [95% CI, 7.7% – 38.6%]	2.8 mo [95% CI, 1.7 ‐ 5.4 mo]	Not reported
Traina et al 2018^29^	ER negative PgR negative HER‐2 negative	Enzalutamide	118 (ITT) 78 (Eval.)	ITT > 0% Eval. ≥10%	ITT: 7/118 Eval.: 6/78	At 24 wk: ITT: 20% [95% CI, 14% – 29%] Eval.: 28% [95% CI, 19% – 39%]	ITT: 2.9 mo [95% CI, 1.9 ‐ 3.7 mo] Eval.: 3.3 mo [95% CI, 1.9 ‐ 4.1 mo]	ITT: 12.7 mo [95% CI, 8.5 ‐ 16.5 mo] Eval.: 16.5 mo [95% CI, 12.7 ‐ 20.0 mo]

Abbreviations: AR, androgen receptor; CBR, clinical benefit rate; CI, confidence interval; ER, estrogen receptor; Eval., evaluable subgroup; HER‐2, human epidermal receptor‐2; ITT, intention‐to‐treat population; ORR, objective response rate; OS, overall survival; PFS, progression‐free survival; PgR, progesterone receptor.

These three studies demonstrated that bicalutamide, abiraterone acetate, and enzalutamide had a good safety profile but only weak clinical activity. However, as reported in the abiraterone acetate study, all patients who experienced clinical benefit had a high level of androgen receptor expression (≥90%). This suggests that patients with MABC who have high AR expression may derive real benefit from anti‐androgen therapy.

Given the similarities between cutaneous and breast apocrine carcinoma, and based upon the high level of AR expression (100%) in our patient's case, we extrapolated that anti‐androgen therapy would be beneficial. We are pleased to report that this treatment was indeed successful.

## 4 CONCLUSION

Primary cutaneous apocrine adenocarcinoma is an extremely rare tumor for which there is no treatment consensus in the inoperable or metastatic setting. In distant metastases with androgen receptor expression, anti‐androgen therapy may have activity as illustrated by this case report.

## CONFLICT OF INTEREST

None declared.

## AUTHOR CONTRIBUTIONS

All authors reviewed and revised the manuscript. Pr Marc Hamoir and Pr Sandra Schmitz provided all the details about the surgical treatment. Dr Pascal Van Eeckhout described the histopathological features and provided the pictures of the biopsies. Dr Philippe D’Abadie described the evolution on ^18^FDG‐PET‐CT and provided the pictures of ^18^FDG‐PET‐CT. Pr Thierry Duprez described the evolution on MRI and provided the pictures of MRI. Pr Jean‐Pascal Machiels reviewed, revised, and coordinated the writing of the manuscript at each step, and provided updates of the patient's evolution at every visit.

## ETHICAL STATEMENT

The patient provided consent for the publication of his case and the use of images from his medical record.
